# Potential ecological risk assessment of heavy metals (trace elements) in coastal soils of southwest Iran

**DOI:** 10.3389/fpubh.2022.889130

**Published:** 2022-09-07

**Authors:** Ebtessam Hamid, Khoshnaz Payandeh, Mohammad Tahsin Karimi Nezhad, Naghmeh Saadati

**Affiliations:** ^1^Department of Soil Science, Ahvaz Branch, Islamic Azad University, Ahvaz, Iran; ^2^Department of Agriculture and Plant Breeding, Sanandaj Branch, Islamic Azad University, Sanandaj, Iran

**Keywords:** pollution load index (PI), toxic elements, Iran, ecological risk index (ERI), heavy metals

## Abstract

Heavy metal pollution has become one of the most important threats that can endanger the health of animals, the environment, and humans. The present study was performed to investigate the potential ecological risk (PER) of heavy metals [zinc (Zn), copper (Cu), cobalt (Co), molybdenum (Mo), manganese (Mn), and selenium (Se)] in the coastal soils of southwest Iran in 2019. The samples were collected from six soil sites and three depth intervals (0–15, 15–30, and 30–45 cm) among bare and vegetated coastal soils. The soil samples to study the soil properties (soil grain size, pH, EC, and soil organic carbon) and metal contamination were taken from soil (36 samples), water (6 samples), and plants (24 samples). The soil ecological risk (ER), the pollution load index (PLI), contamination degree (Cdeg), modified contamination degree (mCdeg) for heavy metal contamination in the soil, and enrichment factor (EF index) indicate the origin of metals entering the environment, and hence these parameters were investigated. The results of this study showed that the levels of Zn, Cu, Co, Mn, Se, and Mo were in the range of low-risk contaminants in this region. According to the results of the study, the risk index (RI) for metals was in the range of 1.296–3.845, which is much lower than 150, and therefore the ecological risk potential calculated in this study was in the low-risk category for toxic elements. Based on the results, it was found that agricultural, industrial, and human activities played an effective role in the accumulation of Zn, Cu, Co, Se, and Mo in the soil. In addition, the main source of Mn metal is believed to be natural due to geological activities in the region.

## Introduction

Human activities, such as industrial, agricultural, and fuel consumption, produce a high level of contaminants (heavy metals, polyaromatic hydrocarbons, and organic and inorganic pollutants) that can threaten the environment (aquatic, soil, and air), animals, and public health (1, 2). Heavy metals (HMs), such as zinc (Zn), copper (Cu), cobalt (Co), manganese (Mn), selenium (Se), and molybdenum (Mo), are trace elements that, if introduced into the living environments of beasts and humans, can cause numerous complications (3–5). Heavy metals have serious complications, including mutagenic effects, carcinogenic effects, toxicity, accumulation in adipose tissue, and long shelf life (6–8). The main sources of these heavy metals are the natural origin (weathering of soils and rocks, environmental erosion, forest fires, and volcanic eruptions) and the anthropogenic origin (the discharge of municipal and industrial effluents, discharge of agricultural waste, mining activities, and urban runoff) (9–13). These activities prevent heavy metals from entering human food chains and accumulating in plants and vegetables, which can lead to serious health consequences, such as carcinogenic risk, genotoxicity, cardiovascular diseases, skin allergies, thrombosis symptoms, and neurological diseases (14, 15). According to the results of different studies, heavy metals gradually accumulate in the body. Accumulation in the adipose tissue, muscles, and bones, and non-biodegradable properties are the most important characteristics of heavy metals (16–21).

Wetlands are aquatic ecosystems that support native biomass with valuable hydro-climatic and ecological capabilities that play a pivotal role in refining the environment and economic life of each region (19, 22). Proper protection and maintenance of wetlands are essential to take advantage of these properties by preventing the production of micro-streams in some regions (19, 22). Wetlands are sites that have a high potential for food storage and play a role in maintaining food cycles for primary producers. Wetlands can be natural, artificial, or a mixture of both. They are not able to withstand the impact and pressure due to increasing human activities and stagnation, and hence are rapidly diminishing. Investigating the potential of wetlands and designing some plans on the margins would help us to identify the issues we would be facing in the ecosystem of a wetland area, and a lack of coordination of a plan on the margins of a wetland would have critical consequences for a wetland area (23, 24).

Heavy metals are one of the important and dangerous compounds that can cause osteoporosis (25, 26). The toxic element that is widely known to have toxic effects on bones is cadmium (25). The main sources of emissions into the environment include tobacco, cell phone batteries, fertilizers, and industrial wastes (26–28). Osteoporosis is a systemic disease that has spread widely throughout the world. Research has shown that about one-third of women and one-tenth of men aged 50 years and above suffer from osteoporosis, the consequences of which can be referred to as atheromatous fracture (26). In the research conducted on osteoporosis, environmental factors are often recognized as intrinsic factors (29). The bone acts as a reservoir for ingested heavy metals and following exposure to external environmental factors (30). Heavy metals, such as lead, cadmium, mercury, and aluminum, accumulate in the skeleton and bind to the calcium in hydroxyapatite, which causes a direct reduction, while indirectly, the concentration of calcium increases the bone-resorbing cells and decreases the bone-forming cells, thus leading to osteoporosis due to decreasing bone-forming cells and increasing bone-resorbing cells (30–32).

In recent years, the presence of high amounts of heavy metals and contamination of water and soil resources can seriously threaten the health of Iranian citizens (33). In Iran, the results of different studies showed that environmental pollution (from industrial areas or agricultural fertilizers) and the presence of heavy metals in the environment and food cycle can be important factors that significantly impact the health of the citizens (34, 35). Khuzestan Province has many permanent and seasonal rivers, wetlands, fertile soil, water, and land roads, and is one of the main agricultural poles of Iran (35). Therefore, the presence of heavy metals in the wastewater entering the river, wetlands, and lands can be considered as a potential cause of pollution and danger (36). Shadegan wetland has an important role in the economy of the region, biodiversity, and habitat diversity, modulating the climate, water absorption, soluble elements, and flood control in southwestern Iran, especially in the Khuzestan Plain (37, 38). Shadegan wetlands have been affected by climate change, and many of its areas have dried up over the years, which had a devastating impact on its environment. For instance, the wetland has suffered severe drought, sewage entry, oil pollution, decreasing water levels, increasing heavy metals, and the accumulation of large quantities of non-degradable waste, triggering problems in the natural ecosystem of this wetland. Specifically, the main problem of Shadegan wetland is the entry of wastewater produced by the steel industry and sugar cane industry into the wetland (39, 40). This wetland covers an area of approximately 4,000 ha. Shadegan wetland is accessible through the highway from Ahvaz to Abadan located on the west side of Shadegan wetland (37). The annual waterfall to the Shadegan wetland from the Jarahi River is 2 million m^3^, but this amount has considerably decreased due to recent drought conditions. In recent years, life in Shadegan wetland has been threatened due to the entry of sugarcane fields, effluents of Malh and Bahreh streams, cane drainage, and the entry of wastewater (41).

The aim of this study was to evaluate the potential ecological risk of heavy metals (zinc, copper, molybdenum, cobalt, manganese, and selenium) in the coastal soils of southwest Iran (Shadegan wetland) in 2019.

## Materials and methods

### Study area

**Figure 1:**
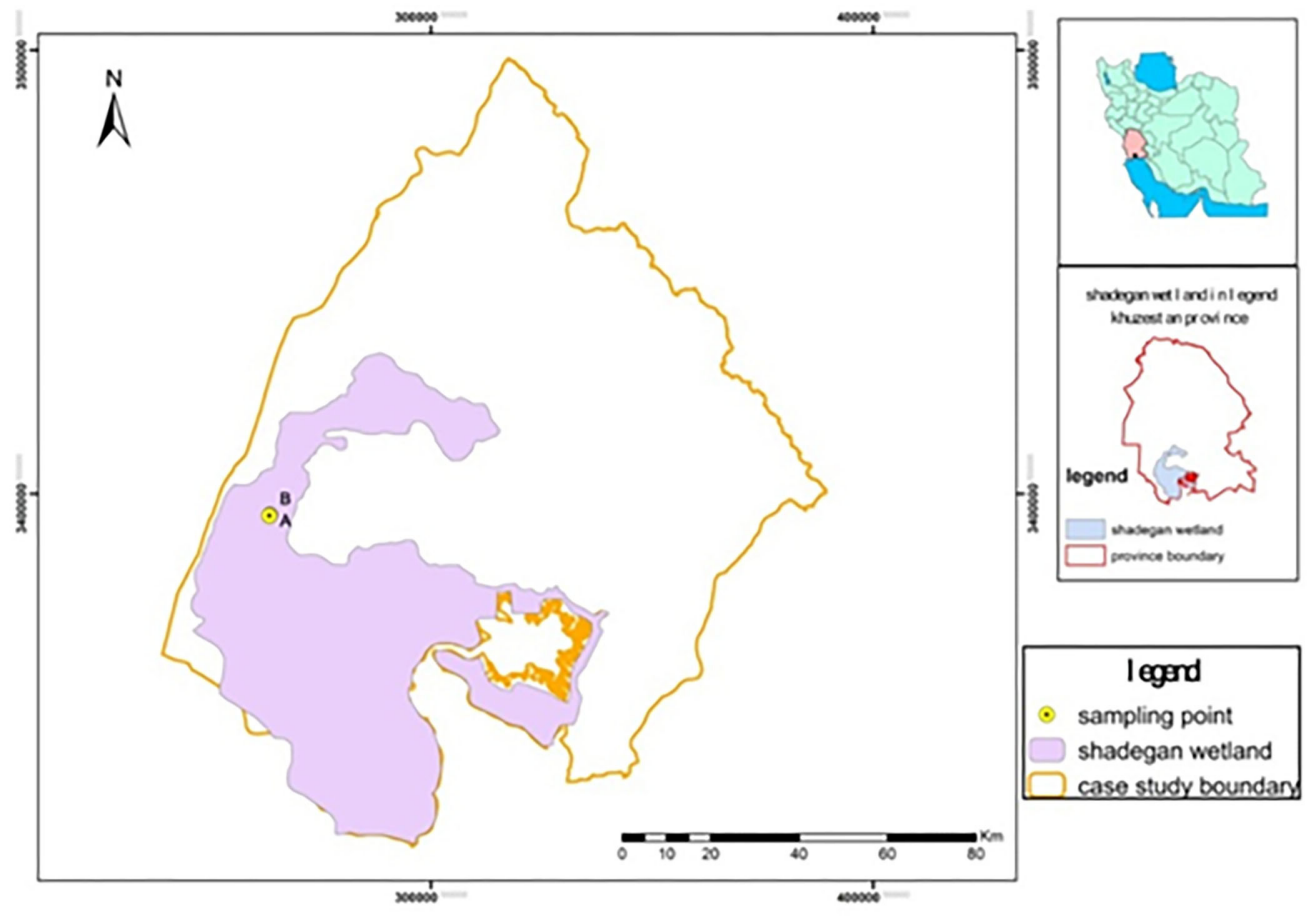
The geographical location of Shadegan coastal water and soil sampling sites.

Shadegan wetland is located in southwestern Iran and south of Khuzestan Province, between the cities of Shadegan, Abadan, and Mahshahr (48°17' to 48°50' E and 30°17' to 30°58' N) (37, 38, 42). It borders Iraq on the west and the Persian Gulf on the south (Figure 1) (43). Figure 1 shows the study area of Shadegan wetland. The three zones that make up the most important parts of Shadegan wetland are freshwater, tidal-coastal, and saline. Shadegan wetland is classified as brackish to saline and is mainly composed of hard water.

### Sampling strategy

Measurement of the parameters and soil sampling were performed at sites that showed different vegetation types. Samples were collected at sites A (with dominant vegetation of wetland shoreline) and B (with bare soils of wetland shoreline) based on the standard methods provided (44, 45). To compare sites A and B to a depth of 45 cm, soil samples from each site were collected from a triangular plot containing three sampling points 200 m apart.

The soil sampling strategy was performed according to the American Society for Testing and Materials D2488 standard at a depth of 45 cm in each station with three replications in summer and winter. The collected soil samples were divided into 15-cm depth intervals. Samples were taken at three depths of 0–15, 15–30, and 30–45 cm, and then transported to the laboratory in a zippered plastic bag. Due to the heterogeneity of the soil environment, selecting only one point at a sampling station in natural areas that are highly influenced by environmental factors causes the sampling station to lose accuracy and increase the variance between replicates. To increase the accuracy of the study, an attempt was made to follow a triangular or square sampling configuration until the combination of samples created an index sample representing an area of 1–10 square meters. Additionally, since wet coastal soils were sampled (shoreline), the increasing temperature in summer and the increased evaporation from the wetland surface may reduce the water level by several centimeters and dry the previous sample site in winter. Therefore, according to the sampling strategy of soils, sampling at a few centimeters from the previous location near the water surface was carried out as a triangular sampling strategy, which resulted in minimizing the error (46).

### Sample analysis

Soil texture and pH value were determined by the hydrometric method and pH meter, respectively. The soil samples were placed in an oven at 65 °C for 120,150 min to reach a constant weight until near drying. After cooling the samples, the wetting method was used to digest the samples by pouring 0.5 g of the sample into a 250 ml balloon and adding 25 ml of concentrated sulfuric acid and 20 ml of 7 M nitric acid. The soil samples were digested with hydrochloric acid (HCl) and nitric acid (HNO_3_) in a ratio of 3:1 (HNO_3_: HCl). After cooling the samples, 20 ml of a 1:1 mixture of concentrated nitric acid and concentrated perchloric acid was gently added from the top. In the next stage, the mixture was heated until the white vapors of acid completely disappeared. The mixture was cooled, and 10 ml of distilled water was added slowly while the balloon was rotated. After heating the mixture for about 100 min, a clear solution was obtained, which was then cooled and transferred to a 100 ml balloon (10, 47, 48).

Heavy metals were evaluated by inductively coupled plasma spectroscopy (ICP-MS). ICP-MS model Varian 710-ES was used to characterize the heavy metals (47). Quality assessment and quality control of the device were performed using reference and standard materials (SRM-2710 and SRM 2710a for Se), respectively. The results indicated that the recovery for the analyzed metals was 93.2–101.8%. The potential ecological risk index (ERI), the pollution load index (PLI), contamination degree (Cdeg), modified contamination degree (mCdeg) for heavy metal contamination in the soil, and enrichment factor (EF index) indicate the origin of metals entering the environment, which were investigated in this study.

### Contamination factor

The contamination factor (CF) was used to evaluate heavy metal contamination (Equation 1).


(1) $$\begin{array}{l} C_{\text{f}}\;=\;C_{\text{o}}\;÷\;C_{\text{n}} \end{array}$$

In the equation, Cn represents the concentration of each element in the sediment, and Co is the average concentration of each element in the shale (Supplementary Table S1) (49, 50).

### Contamination degree

The sum of the contamination factors for the investigated elements represents the degree of contamination (Cdeg) obtained from the following equation (2) (Supplementary Table S1) (50).


(2) $$\begin{array}{l} Cdeg=\Sigma Cf \end{array}$$

Supplementary Table S2 shows that due to the limitations of the contamination degree equation (Cdeg), the modified contamination degree was introduced which can be calculated from the following equation (3): (51).


(3) $$\begin{array}{l} mCdeg=\Sigma Cf/n \end{array}$$

### Ecological risk assessment

In this study, the following relationships were used to assess the ecological risk and environmental risk potential (RI) of coastal soils of the wetland (50).


(4) $$\begin{array}{l} ERI=TR\times CF \end{array}$$

(5) $$\begin{array}{l} RI=\sum ERI \end{array}$$

where CF is the pollution factor, ER indicates the ecological risk of each element studied, and RI represents the sum of the elements. The value of TR indicates the toxicity of heavy metals (50). Supplementary Table S3 expresses ecological risk for each element as a five-level classification: ER = 40, low risk; 80 > ER > 40, medium risk; 160 > ER > 80, significant risk; 320 > ER > 160, high risk; and ER = 320, very high risk. For the analysis of risk index (RI), four categories are as follows: RI < 150, low risk; 300 > RI ≥ 150, medium risk; 600 > RI ≥ 300, significant risk; and RI ≥ 600, very high risk (50).

### Pollution load index

The pollution load index is evaluated to determine the level of contamination of heavy metals. The values of the PLI range from zero (non-contaminated) to 10 (highly contaminated). Typically, values of <1 indicate non-contamination, and values of >1 indicate contamination with heavy metals. This index can be calculated by multiplying the indices of heavy metal pollution by employing the following equation (6) (50, 52).


(6) $$\text{PLI}=\sqrt[{\text{n}}]{\text{CFn}1\;\times \;\text{CFn}2\;\times \;\ldots \ldots \;\times \;\text{CFni}}$$

In this equation, CF is the contamination factor obtained from the contamination factor for each metal.

### Enrichment factor

The determination of the EF indicates the level of contamination of metals in the soil, which is a useful index for separating the natural and human sources of metals from each other. To calculate the metal EF, the normalized metal (EF) and the background value of the metals should be determined. In several studies, iron and aluminum, which have the lowest levels of human contamination, have been used as normalizers. In this paper, the iron element was utilized to separate the human component from the natural one. The EF for each metal was calculated based on the ratio between the normalized element and the background value of the elements (53). This index is obtained by Equation 7:


(7) $$\begin{array}{l} EF={\left({\text{Cx}}_{\text{Metal}}/Fe\right)}_{\text{Sample}}\;÷\;{\left({\text{Cref}}_{\text{Metal}}/Fe\right)}_{\text{Background}} \end{array}$$

where *Cref* is the concentration of the reference element in the sample, and Cx is the concentration of the *element considered* in the sample. A geological origin is the most important factor in determining the reference element (53). The reference element in determining the enrichment factor is an element that has a purely geological origin (53). In this research, the iron *(*Fe) element was used as a reference metal. Supplementary Table S4 also presents the classification of samples according to the enrichment factor.

### Statistical analysis

Data analysis was used to produce descriptive statistics for the soil pollution indices. The concentration of the heavy metal was analyzed using SPSS version 25 and SigmaPlot statistical software. Multivariate analyses, principal component analysis (PCA), correlation matrix, and general linear model (GLM) analysis were performed. Excel software was employed to draw charts and tables. One-way analysis of variance (ANOVA), two-way ANOVA, and Tukey's HSD test were performed to compare the significant differences among the groups.

## Results

In this study, results indicated that soil texture in the coastal soils of Shadegan wetland was determined as loamy, sandy-loamy, and clayey-loamy-sandy. The percentage of sand particles in coastal soils, without vegetation and with vegetation in Shadegan wetland at different depths, was higher than clay and silt percentages. The highest soil acidity (pH~7.40) was obtained at a depth of 30–45 cm of non-vegetated soil, and the lowest soil acidity (6.86) was observed at a depth of 0–15 cm (Table 1). In the vegetated soils, the pH value (7.36) at the depth of 30–45 cm was greater when compared to the other studied depth intervals, which were lower compared to the pH at the depth of 0-15 cm in non-vegetated sites (Table 1). The highest and lowest electrical conductivity (EC) values were obtained as 29.80 and 1.88 ds.m^−1^ at the depths of 0–15 and 30–45 cm, respectively (Table 1). Electrical conductivity values at the depth of 0–15 cm in the soil with no vegetation cover were observed to be higher than the other studied depths. In naturally vegetated soils at the first and second stations at the depths of 30–45 cm, the above-mentioned parameter was higher when compared to the depths of 0–15 and 15–30 cm (Table 1).

**Table 1:** Analysis of variance (ANOVA) for metal concentrations in coastal soil and water samples of Shadegan wetland.

**Variable**	**Source**	**Type III sum of squares**	**Degree of freedom**	**Mean of squares**	***F*-value**	***P*-value**
Zn	S	1.590	1	1.590	7.546	0.011^*^
	VC	1.611	1	1.611	7.646	0.011^*^
	D	2.846	2	1.423	6.754	0.005^**^
	S × VC	2.158	1	2.158	10.245	0.004^**^
	S × D	0.327	2	0.163	0.776	0.472ns
	VC × D	0.069	2	0.035	0.164	0.850ns
	S × VC × D	1.110	2	0.555	2.634	0.092ns
Cu	S	3.133	1	3.133	21.053	0.000^***^
	VC	0.001	1	0.001	0.005	0.945ns
	D	0.442	2	0.221	1.487	0.246ns
	S × VC	0.240	1	0.240	1.613	0.216ns
	S × D	0.674	2	0.337	2.265	0.126ns
	VC × D	0.118	2	0.059	0.396	0.677ns
	S × VC × D	0.143	2	0.071	0.479	0.625ns
Co	S	3.650	1	3.650	1,514.859	0.000^***^
	VC	0.001	1	0.001	0.475	0.497ns
	D	0.023	2	0.012	4.776	0.018^*^
	S × VC	0.001	1	0.001	0.386	0.540ns
	S × D	0.017	2	0.008	3.427	0.049^*^
	VC × D	0.001	2	0.001	0.304	0.741ns
	S × VC × D	0.004	2	0.002	0.925	0.410ns
Mn	S	7.508	1	7.508	2.311	0.142ns
	VC	6.167	1	6.167	1.898	0.181ns
	D	11.997	2	5.999	1.846	0.180ns
	S × VC	0.427	1	0.427	0.131	0.720ns
	S × D	9.616	2	4.808	1.480	0.248ns
	VC × D	0.691	2	0.345	0.106	0.900ns
	S × VC × D	3.033	2	1.516	0.467	0.633ns
Se	S	0.036	1	0.036	0.077	0.784ns
	VC	0.191	1	0.191	0.408	0.529ns
	D	0.819	2	0.410	0.876	0.429ns
	S × VC	6.674	1	6.674	14.270	0.001^**^
	S × D	0.336	2	0.168	0.359	0.702ns
	VC × D	0.335	2	0.168	0.358	0.703ns
	S × VC × D	0.121	2	0.060	0.129	0.879ns
Zn-water^a^	S	0.025	1	0.025	8.642	0.042^*^
Cu-water^a^	S	0.014	1	0.014	9.894	0.035^*^
Co-water^a^	S	1.017	1	1.017	10.973	0.030^*^
Mn-water^a^	S	0.000	1	0.000	0.000	1.000ns
Se-water^a^	S	0.000	1	0.000	0.000	0.992ns

S, season; VC, vegetation cover; D, soil depth; ns, not significant.

a one-way ANOVA results.

*** P < 0.001;

** P < 0.01;

* P < 0.05.

The ANOVA results (Table 1) indicated that seasonal variation (S), vegetation cover (VC), and soil depth (D) had significant effects on the Zn content at the significance values of *P* < 0.05 and *P* < 0.01. Seasonality (*P* < 0.001) and soil depth (*P* < 0.05) significantly affected the Co concentrations in the coastal soils of Shadegan wetland, whereas Cu concentrations were remarkably (*P* < 0.001) influenced only by the seasonal variation. Season, plant cover, and soil depth did not show a significant effect on the amount of Mn and Se.

**Figure 2:**
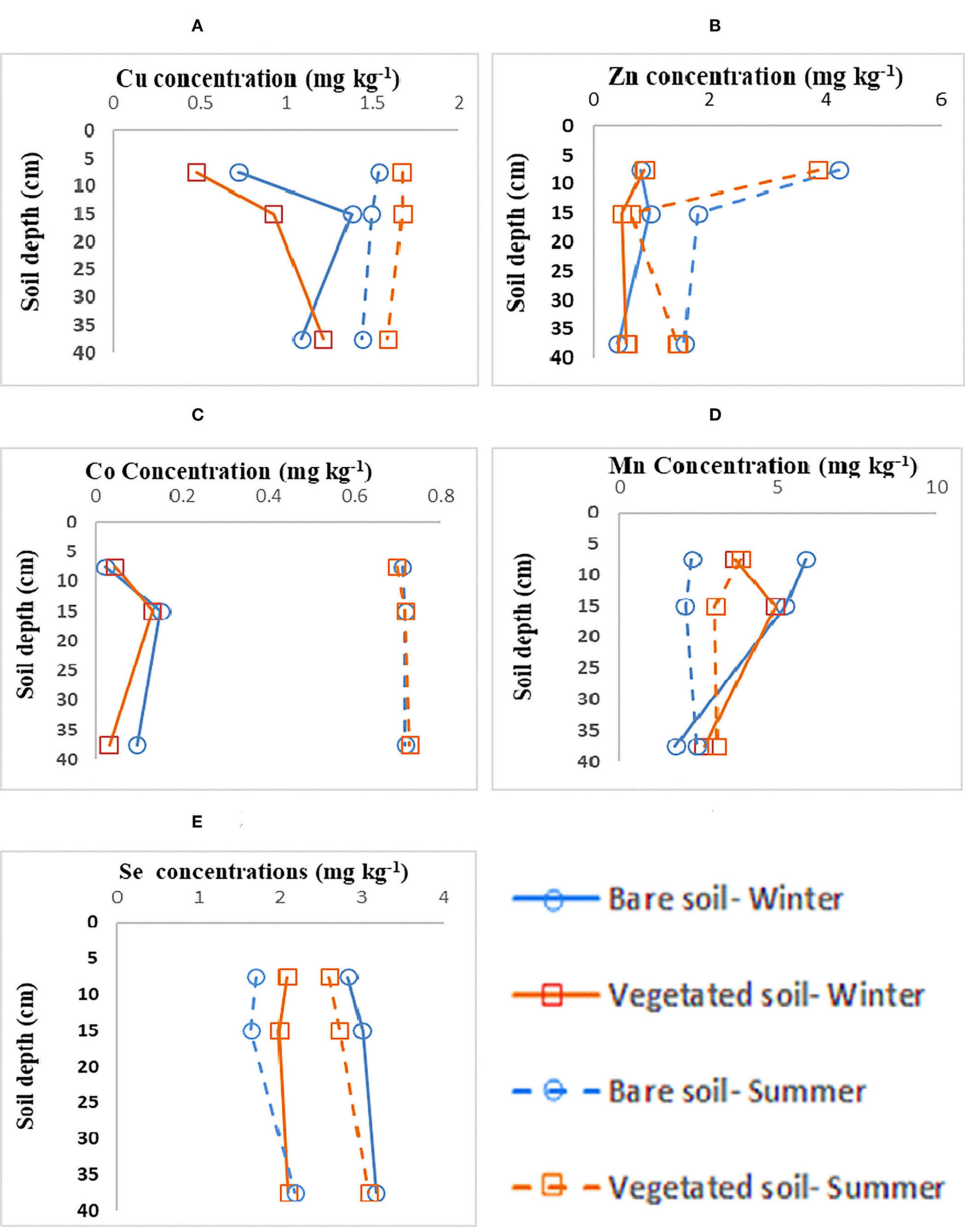
**(A–E)** Metal depth profiles in Shadegan wetland hydric coastal soils over two seasons, three depth intervals, and two vegetation cover levels.

Regarding the relationship between the season and the vegetation cover level, a significant effect was observed on the Zn and Se content as well as soil depth on Co values. The comparison between the means for SOC and stable C isotopic composition showed that the highest amounts of SOC and the smallest δ^13^C values at all three depths were observed in the soils developed over foliated metamorphic (FM) bedrock lithology (Figures 2B,C,E).

The depth profile of the studied metals demonstrated different fluctuations (Figures 2A–E) with variations in season and vegetation cover. For Zn, Cu, and Co, higher concentrations were observed in summer-collected samples, while concerning Mn, winter-collected bare soils had slightly higher concentrations followed by vegetated soils (Figures 2A–E). These results support our hypotheses that site-specific factors (lithology and soil depth) significantly influence the SOC storage and its turnover across the studied geologic gradient.

Concerning the significant effect of seasonal variation on the concentrations of some of the studied metals (Supplementary Table S5), multivariate analyses were performed for the winter and summer datasets separately. The results of the Spearman rank correlation coefficient between metals and soil properties (grain size, pH, SOC, and EC) in winter soil datasets are shown in **Table 6**. Clay and sand fractions in the winter-collected soil samples displayed a significant negative correlation with silt particles at *P* < 0.001. In the soils that were collected in the winter, there was no significant link between the amount of metal and the size of the soil particles (Supplementary Table S5).

Soil organic carbon (SOC) revealed a significant negative correlation with Se (*P* < 0.05, *r* = −0.479). The significant negative correlation between Se and organic fraction showed the possible oxidation state of Se, as selenide (Se^−2^) with Eh values < 200 mv (54), but no affinity of this metal for the organic compounds was observed in the winter-collected soils.

Soil pH dictated a strong negative correlation with Zn (−0.504, *P* < 0.05) and Mn (−0.676, *P* < 0.01) concentrations. Inter-element relationships can provide information on metal sources and behavior. Cu and Co, as well as Mn and Zn, are strongly correlated with each other at *P* < 0.01 and *P* < 0.05. The results revealed that these elements had similar geochemical behavior (Supplementary Table S5).

Unlike the winter dataset, the Spearman rank correlation coefficient between clay and sand grain sizes in the summer dataset had significant correlations with Zn at *P* < 0.01 (Supplementary Table S6). The significant correlation between pH and metals was observed only for Co (r =0.492) at *P* < 0.05. A significant negative correlation at *P* < 0.01 was found between the elemental pair Zn–Se (-0.697), suggesting different geochemical behaviors for these elements (Supplementary Table S6).

### Principal component analysis

To better infer the relationship between soil characteristics, principal component analysis (PCA) was applied to both winter and summer datasets. Multivariate analysis (principal component analysis/PCA) was employed to further investigate the relationships between the trace elements and edaphic properties in the studied hydric soils. The results of PCA for metals and soil properties for winter-collected soil samples are presented in **Table 8**. The findings for the component loaded with varimax rotation, along with the eigenvalues and commonalities, indicated that four components account for 80% of the total variance of the soil dataset. The first component (PC1), which explained 28% of the total variance, includes Zn, Mn, pH, and EC. The strong and positive loading values of Zn (0.751), Mn (0.855), and EC (0.718) suggest the strong relationship of these elements with the soluble ions (electrical conductivity/EC) of the soil (Table 2).

**Table 2:** Rotated component matrix for winter- and summer-collected soil datasets of the hydric soils of Shadegan wetland.

**Variable**	**Winter dataset**			
	**PC1**	**PC2**	**PC3**	**PC4**
Zn	**0.751**	−0.291	−0.237	−0.082
Cu	0.021	0.307	**0.704**	0.396
Co	−0.026	0.129	**0.932**	−0.060
Mn	**0.855**	−0.240	0.223	−0.095
Se	−0.029	**0.812**	0.175	−0.018
Silt	0.166	0.245	−0.216	–**0.909**
Clay	0.108	0.176	−0.092	**0.919**
Sand	−0.257	–0.538	0.380	**0.542**
pH	–**0.866**	−0.076	−0.056	−0.093
EC	**0.718**	0.431	−0.307	−0.112
SOC	0.175	–**0.767**	−0.185	−0.007
Eigenvalue	3.122	2.255	2.130	1.256
% of variance	28.382	20.504	19.368	11.422
% of cumulative	28.382	48.886	68.254	79.676
**Variable**	**Sumer dataset**			
	**PC1**	**PC2**	**PC3**	**PC4**
Zn	−0.910	0.107	0.091	−0.032
Cu	0.120	0.169	0.890	0.025
Co	−0.006	0.386	0.381	0.734
Mn	−0.104	0.916	0.197	−0.092
Se	0.839	0.107	−0.089	−0.061
Silt	0.590	0.049	0.245	−0.160
Clay	0.704	–0.514	0.120	−0.011
Sand	−0.751	0.458	−0.127	0.270
pH	−0.224	−0.222	0.022	0.881
EC	−0.215	−0.168	0.533	−0.718
SOC	0.471	0.381	0.200	−0.693
Eigenvalue	3.783	2.295	1.695	1.080
% of variance	34.390	20.864	15.406	9.819
% of cumulative	34.390	55.254	70.660	80.479

Extraction method: a principal component analysis.

Rotation method: Varimax with Kaiser normalization.

The second component (PC2) is the second strongest factor, showing 20.5% of the total variability. This component includes Se (0.812), SOC (-0.767), and sand with a medium negative loading value of −0.538. The third component (PC3) involves Cu (0.704) and Co (0.932) and explains 19.37% of the total variability. The fourth component (PC4) includes silt (-0.909), clay (0.919), and sand (0.542), explaining 11.4% of the total variability.

Regarding summer-collected soils, four principal components (with a sum of squares of loading >1) explain 80.48% of the total variance of the dataset (Table 2). Compared with the PCA results of the winter dataset, the loading matrix of the components from the summer dataset revealed different groupings. The first component (PC1) groups Zn, Se, silt, clay, and sand (accounting for 34.39% of total variance), the second component (PC2) groups Mn and clay with a medium loading value of −0.514 (explained variance of 20.86%), and the third component (PC3) is heavily loaded only by Cu (0.890) (with 15.41% explained variance). The last component (PC4) (explained variance of 9.82%) indicated a negative correlation between Mn and clay and a positive correlation between Co and pH (Table 2).

Table 3 shows the results of ANOVA analysis for metal concentration in plant materials. It was found that seasonal variation had a significant effect on the Cu and Co concentrations in both *Juncus acutus* and *Hammada salicornica* plant species, as well as Zn and Mn content only in the *Hammada salicornica* plant type (Table 3). Zinc and Mn levels were significantly different between above- and below-ground tissues for both plant species. For Se, shoot vs. root concentration was significantly different only for the *Juncus acutus* plant type. No substantial difference was observed in Cu and Co levels between the above- and below-ground biomass of the two plant species. The interaction between seasonal variation and part of the plant (shoot vs. root) was found to be significantly different only for Se concentration in *Hammada salicornica* plant species (Table 3).

**Table 3:** Analysis of variance (ANOVA) for metal concentrations in coastal plant materials of the Shadegan wetland.

**Variable**	**Source**	**Type III sum of squares**	**Degree of freedom**	**Mean of squares**	***F*-value**	***P*-value**
**Zn** (*Juncus acutus***)**	S	0.124	1	0.124	4.703	0.062ns
	P	0.208	1	0.208	7.888	0.023^*^
	S × P	0.002	1	0.002	0.062	0.810ns
**Zn** (*Hammada salicornica***)**	S	0.811	1	0.811	10.712	0.011^*^
	P	0.644	1	0.644	8.505	0.019^*^
	S × P	0.062	1	0.062	0.814	0.393ns
**Cu** (*Juncus acutus***)**	S	1.038	1	1.038	15.922	0.004^**^
	P	0.310	1	0.310	4.760	0.061ns
	S × P	0.055	1	0.055	0.838	0.387ns
**Cu** (*Hammada salicornica***)**	S	2.001	1	2.001	142.577	0.000^***^
	P	0.028	1	0.028	1.998	0.195ns
	S × P	0.012	1	0.012	0.857	0.382ns
**Co (** *Juncus acutus* **)**	S	1.153	1	1.153	1,976.914	0.000^***^
	P	0.003	1	0.003	4.629	0.064ns
	S × P	0.000	1	0.000	0.514	0.494ns
**Co** (*Hammada salicornica***)**	S	1.123	1	1.123	1,246.761	0.000^***^
	P	0.004	1	0.004	4.937	0.057ns
	S × P	0.001	1	0.001	1.588	0.243ns
**Mn** (*Juncus acutus***)**	S	0.137	1	0.137	0.651	0.443ns
	P	1.841	1	1.841	8.781	0.018^*^
	S × P	0.077	1	0.077	0.366	0.562ns
**Mn** (*Hammada salicornica***)**	S	6.206	1	6.206	131.701	0.000^***^
	P	1.261	1	1.261	26.759	0.001^***^
	S × P	0.009	1	0.009	0.193	0.672ns
**Se (** *Juncus acutus* **)**	S	0.568	1	0.568	0.274	0.615ns
	P	24.912	1	24.912	12.011	0.008^**^
	S × P	0.445	1	0.445	0.214	0.656ns
**Se (** *Hammada salicornica* **)**	S	0.037	1	0.037	0.248	0.632ns
	P	8.333E-6	1	8.333E-6	0.000	0.994ns
	S × P	13.167	1	13.167	87.445	0.000^***^

S, season; P, plant part (above- vs. below-ground biomass); ns, not significant.

*** P < 0.001;

** P < 0.01;

* P < 0.05.

**Figure 3:**
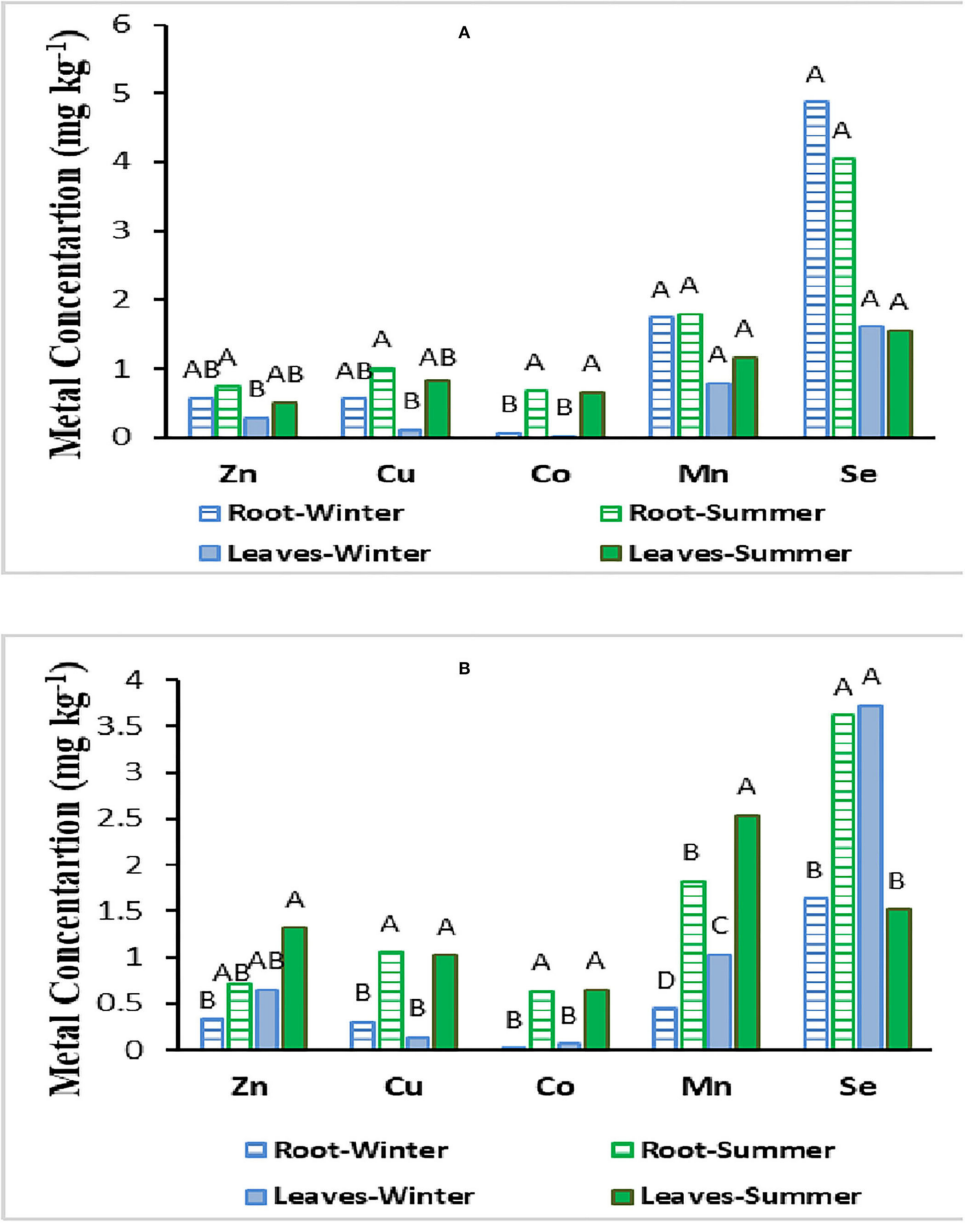
Metal concentrations in above- and below-ground biomass of *Juncus acutus* **(A)** and *Hammada salicornica* **(B)** plant species collected in winter and summer of 2019.

As could be seen in Figure 3, metal concentrations were slightly higher in the below-ground biomass of *Juncus acutus* plant species even though there were no significant differences between shoot and root metal content (Figure 3). On the contrary, in *Hammada salicornica* above-ground biomass, slightly higher metal accumulation was observed compared to the below-ground part. Se concentration followed by Mn concentration was higher compared to those of other studied metals in both shoot and root biomass of the studied plant species (Figure 3).

### Contamination indices

The CF of Se was higher in winter and summer in all the studied stations when compared to Zn, Cu, Co, Mn, and Mo. In general, the highest level of CF was 3.666 for Se in the third station without vegetation cover in winter, and the lowest value was 0.0005 for Co in the second station with vegetation cover in winter. The value range of CF for Zn was 0.002–0.050, and the highest value was obtained for Zn at the third station with vegetation cover in summer, and the lowest was obtained at the third station in winter (Table 4). The highest and lowest levels of CF for Cu were 0.043 and 0.015, respectively, at the third station in summer and the second station in winter. For Co, the highest CF was 0.038 at the third station with vegetation cover in summer and the lowest value was 0.0005, which was obtained at the second station with vegetation cover (Table 4). The CF for Mn ranged from 0.002 to 0.007. The highest and lowest values of contamination factor for Se were obtained in the third and first stations in winter, with 3.666 and 1.01, respectively. For Mo, the highest CF was observed to be 0.280 in summer at the first station, and the lowest was 0.019 at the second and third stations in winter (Table 4).

**Table 4:** The heavy metal contamination factor (CF) in the coastal Shadegan soils in winter of 2019.

**Season**	**Heavy Metal**	**Without Vegetation**			**With Vegetation**		
		**First station**	**Second station**	**Third station**	**First station**	**Second station**	**Third Station**
**Winter**	Zn	0.008	0.008	0.006	0.11	0.005	0.002
	Cu	0.019	0.026	0.025	0.020	0.015	0.22
	Co	0.003	0.006	0.003	0.004	0.0005	0.005
	Mn	0.002	0.005	0.007	0.007	0.003	0.002
	Se	1.777	2.870	3.666	1.101	2.175	2.407
	Mo	0.153	0.203	0.138	0.153	0.019	0.019
**Summer**	Zn	0.032	0.035	0.011	0.006	0.007	0.050
	Cu	0.040	0.020	0.038	0.032	0.033	0.043
	Co	0.036	0.037	0.037	0.036	0.036	0.038
	Mn	0.003	0.003	0.002	0.003	0.002	0.006
	Se	2.083	1.611	2.148	2.092	3.194	2.601
	Mo	0.0280	0.173	0.157	0.023	0.053	0.023

In general, the mean total contamination degree (Cdeg) of Zn, Cu, Co, Mn, Se, and Mo was 0.015, 0.044, 0.020, 0.003, 2.310, and 0.116, respectively. According to the results, the pattern for Cdeg values was as follows: Se > Mo > Cu > Co > Zn >Mn (Table 5).

**Table 5:** Range of contamination degree (Cdeg), modified contamination degree (mCdeg), and pollution load indices in coastal Shadegan soils (winter and summer of 2019).

**Index**	**Season**	**Without Vegetation**			**With Vegetation**		
		**First station**	**Second station**	**Third station**	**First station**	**Second station**	**Third station**
Contamination degree	Winter	1.962	3.118	3.845	1.296	2.222	2.520
	Summer	2.473	1.879	2.393	2.192	3.235	2.761
Modified contamination degree	Winter	0.327	0.519	0.640	0.216	0.370	0.420
	Summer	0.412	0.313	0.398	0.365	0.554	0.460
Pollution load index	Winter	0.085	0.115	0.105	0.100	0.071	0.064
	Summer	0.155	0.140	0.129	0.099	0.112	0.145

The highest Cdeg and mCdeg values of heavy metals were obtained in winter at the third station without vegetation cover, and were found to be 3.845 and 0.640, respectively (Table 5). The lowest levels of Cdeg and mCdeg for heavy metals were 1.296 and 0.221, respectively, in the first station with vegetation cover in winter (Table 5). In summer, the highest Cdeg and mCdeg values were obtained at the second station with vegetation cover, that is, 3.325 and 0.554, respectively, and the lowest Cdeg and mCdeg values for heavy metal at the second station without vegetation cover were 1.872 and 0.313, respectively (Table 5). The highest pollution load index for heavy metals in summer was 0.155 at the first station without vegetation cover, and the lowest pollution load index for heavy metals was observed in winter at the third station with vegetation cover and was equal to 0.064 (Table 5).

The highest ecological risk (ER) for Cu and Co was observed in summer at the third station with vegetation cover and was found to be 0.215 and 0.19, respectively, and for Se was 3.666 in the third station without vegetation cover (Table 6). The highest ER for Zn was observed in summer at the second station without vegetation cover and was found to be 0.035. The ER of Mn ranged from 0.002 to 0.007 (Table 6).

**Table 6:** The ecological risk (ER) of heavy metals in coastal Shadegan soils (winter and summer of 2019).

**Season**	**Heavy Metal**	**Without Vegetation**			**With Vegetation**		
		**First station**	**Second station**	**Third station**	**First station**	**Second station**	**Third station**
Winter	Zn	0.008	0.008	0.006	0.011	0.005	0.002
	Cu	0.095	0.13	0.125	0.1	0.075	0.11
	Co	0.015	0.03	0.015	0.02	0.0025	0.025
	Mn	0.002	0.005	0.007	0.007	0.003	0.002
	Se	1.777	2.870	3.666	1.101	2.175	2.407
	Mo	2.295	3.045	2.07	2.295	0.285	0.285
Summer	Zn	0.032	0.035	0.011	0.006	0.007	0.050
	Cu	0.2	0.1	0.19	0.16	0.165	0.215
	Co	0.18	0.185	0.185	0.18	0.18	0.19
	Mn	0.002	0.003	0.002	0.003	0.002	0.006
	Se	2.083	1.611	02.148	2.092	3.194	2.601
	Mo	4.2	2.595	2.355	0.345	0.795	0.345

For Mo, the highest value of ER was obtained in summer at the first station without vegetation cover, which was equal to 4.2 (Table 6).

The lowest levels of Cu, Co, and Mo were at the second and third stations in winter in the areas with vegetation cover, which were 0.075, 0.0025, and 0.285, respectively. The lowest levels of Se and Zn were also observed in the first and third stations in winter and in the stations with vegetation cover and were 1.101 and 0.002, respectively (Table 6).

The highest mean RI value for heavy metals in the soils of Shadegan wetland was observed in summer at the first station without vegetation cover, which was equal to 6.697 (Table 7). The lowest value of RI for heavy metals was obtained in winter at the second station with vegetation cover, which was found to be 2.545 (Table 7).

**Table 7:** Risk index (RI) value for heavy metals in Shadegan wetland soils (2019).

**Season**	**Without vegetation**			**With vegetation**		
	**First station**	**Second station**	**Third station**	**First station**	**Second station**	**Third Station**
Winter	1.962	3.118	3.845	1.296	2.222	2.250
Summer	2.473	1.879	2.393	1.192	3.235	2.761

The highest values of EF for Zn, Cu, and Co were obtained at the third station with vegetation cover in summer, and were equal to 0.230, 0.217, and 0.206, respectively (Table 8). The highest EF (0.135) for Mo was also observed in summer in the area without vegetation cover at the first station, but the highest value for this index for Mn and Se was observed in winter at the third station without vegetation cover, that is, 0.035 and 0.220, respectively (Table 8). The lowest EF for Zn, which was 0.012, was at the third station with vegetation cover in summer. The lowest value of this index for Mn was obtained in summer at the second station with vegetation cover and at the third station without vegetation cover, which was 0.009. For Cu and Co, the lowest EF was observed at the second station with vegetation cover in winter, which was estimated to be 0.075 and 0.002, respectively (Table 8). For Se and Mo, the lowest values of this index, which were equal to 0.066 and 0.009, respectively, were observed in winter for the areas with vegetation cover at the first and second stations that were equal to 0.066 and 0.009, respectively (Table 8). In general, the mean total EF for Zn, Cu, Co, Mn, Se, and Mo was 0.071, 0.141, 0.109, 0.018, 0.139, and 0.056, respectively. Therefore, according to the results, the pattern of EF values is as follows: Cu > Se > Co > Zn > Mo > Mn.

**Table 8:** Values of enrichment factor for heavy metals in the soils of Shadegan wetland (winter and summer in 2019).

**Season**	**Heavy metal**	**Without vegetation**			**With vegetation**		
		**First station**	**Second station**	**Third station**	**First station**	**Second station**	**Third station**
Winter	Zn	0.039	0.037	0.028	0.054	0.026	0.012
	Cu	0.098	0.130	0.125	0.104	0.075	0.110
	Co	0.016	0.036	0.016	0.022	0.002	0.027
	Mn	0.010	0.026	0.035	0.034	0.014	0.014
	Se	0.106	0.172	0.220	0.066	0.130	0.144
	Mo	0.074	0.098	0.066	0.074	0.009	0.009
Summer	Zn	0.150	0.161	0.547	0.030	0.036	0.230
	Cu	0.203	0.102	0.193	0.162	0.168	0.217
	Co	0.194	0.197	0.206	0.194	0.192	0.206
	Mn	0.010	0.017	0.009	0.016	0.009	0.029
	Se	0.125	0.096	0.129	0.125	19.220	15.654
	Mo	0.135	0.083	0.076	0.011	0.025	0.011

## Discussion

In this study, we investigated the ecological risk assessment and pollution load index of toxic elements (zinc, copper, molybdenum, cobalt, manganese, and selenium) in coastal soils of southwest Iran (Shadegan wetland) in 2019.

Based on the results of this study, the levels of Zn, Cu, Co, Mn, Se, and Mo in the water of Shadegan wetland were higher in summer compared to those in winter. Since Shadegan wetland is located in a hot and dry tropical region, evaporation from the water level is very high in summer and there is no rainfall in the warm seasons, the volume of water entering the wetland reduces, and eventually the water level drops; consequently, the concentration of heavy metals in the water can increase (55). Some studies confirm that the water of rivers and wetlands is polluted with heavy metals in the warm seasons with low rainfall and that the concentrations of these pollutants increase in summer compared to winter. In the summer season, the amount of heavy metals increases due to the low water displacement and high evaporation rate (56).

The highest amount of the studied metals in the water of Shadegan wetland was obtained from the first station. The first station is adjacent to the northern part of the wetland and is the entry point of the Azadegan Fish Wastewater Complex. It is also the entry point of agricultural wastewater from sugarcane and by-products and steel industries. Climate change in the past few years has led to drought conditions in the Shadegan wetland, which has been extremely destructive to the environment. Besides suffering from severe drought, sewage entry, oil pollution, water level reduction, an increasing number of heavy metals, and the accumulation of large amounts of non-degradable waste, these changes have caused problems in the natural ecosystem process of this wetland. The major problem with Shadegan wetland is the entry of 6 million cubic meters of contaminated water into the wetland, of which 40–60,000 cubic meters is associated with the steel industry and 20–25,000 cubic meters is contributed by the sugarcane effluent and domestic sewage.

The amount of Zn measured in the coastal soils of Shadegan wetland was higher in summer than in winter. The highest amount of Zn was observed in winter at the first station and in summer at the third station. Zn is a metal that is widely distributed in the environment; the amount of Zn in the earth is approximately 80 ppm, and in the soil, it varies from 10 to 300 ppm (average of 50 ppm), but in alkaline soils, its amount does not exceed 20 ppm. Furthermore, the Zn content of surface soils is lower than that of underlying soils, confirming the findings of this study (57).

The amount of Zn ranged from 0.15 to 9.41 ppm, which was much lower compared to the global average of this metal, shale, and earth crust (127, 95, and 75 ppm) (20, 58). In addition, the pH of the soil was not acidic like in alkali soils and was nearly neutral. Zn movement in the soil is mainly through diffusion, and the Zn diffusion coefficient in calcareous soils is 50 times lower compared to that in acidic soils, which is a reason for the deficiency of Zn in the calcareous soils of Iran. In addition, other factors, such as the efficiency of soil in terms of Zn-bearing minerals, the existence of alkaline acidity, high calcium carbonate content, abundant bicarbonate in irrigation water, dead soil (soil without bacteria and organic matter), high phosphorus and nitrogen in the soil, and a lack of application of Zn fertilizer, may all have an impact on Zn deficiency in the country's soil ecosystem (9, 59). Zn deficiency in soils has been reported for large areas all around the world. In its comprehensive research, FAO stated that more than 30% of the studied soils are zinc deficient. Certain countries, including Belgium, Malta, Iraq, Turkey, Pakistan, and India, report more severe zinc deficiency than other countries, including Syria, Lebanon, Mexico, and Thailand (60). Generally, Zn deficiency is believed to be severe in soils with high acidity, calcareous soils, sandy soils, sodic soils, soils with low acidity (due to little native Zn in the soil), soils with high and low organic matter content, flooded soils without ventilation, and highly leached soils (61, 62). In Asia, Zn deficiency has been reported to be more severe in arid and semi-arid soils compared to wet and semi-wet soils (63).

Copper in the coastal soils of Shadegan wetland was higher in summer than in winter. The highest amount of Cu was observed in summer at the third station with vegetation cover. Cu metal was obtained in more amounts during the winter and summer in the stations with vegetation cover than in the stations without vegetation cover. The values of Cu in this study ranged from 0.16 to 2.20 ppm compared to the global average, shale, and earth crust (38.9, 45, and 50 ppm), which were much lower (20, 58). Cu metal is utilized in industries for the production of Cu metal pipes, cables, wires, Cu cookware, and so on. It is also used to make birth control pills. Cu is added to drinking water and pools in the form of Cu sulfate (64). Due to human anthropological and industrial activities, it can accumulate in the soil and can be collected by plants (65). The most important sources of Cu for environmental pollution are mining, agricultural, waste, and sewage sludge treatment activities (66).

Soil Co levels were higher in summer than in winter in all the stations without and with vegetation cover. The distribution of Co in the first, second, and third stations was different in the areas with and without vegetation cover. For example, in winter, the content of Co in the third station with vegetation cover was higher than that of the third station without vegetation cover. Meanwhile, at the second station, exactly the opposite situation was observed, which was that the temperature was higher in the station without vegetation cover than in the station with vegetation cover. The distribution of Co in the soil depends on geological factors, human activities, and climate. Different climates are suitable for a specific range of crops and varieties. Mountainous altitudes generally lack dense vegetation cover and are less permeable, so these areas are less prone to low soil Co concentrations (67). The amount of Co in this study was in the range of 0.006–0.080 ppm compared to the global average of this element, shale, and earth crust (1–40, 20, and 25 ppm), which were much lower (20, 58). Various studies have reported the levels of this element in soil (68–70). Chemicals, mineral fertilizers, untreated industrial wastewater, waste, and mineral wastes were major sources of Co and other minerals in soil and water (71).

The Mn content in soil was observed to be higher in winter than in summer, only in the first station without vegetation cover and in the third station with vegetation cover; the amount of this metal was higher in summer than in winter. In winter, the Mn content was higher in the areas without vegetation cover except for the first station. In summer, the Mn content was higher in the areas with vegetation cover except for the second station without vegetation cover. Mn concentration in the soil is largely controlled by redox reactions, but based on the studies showing that variations in seasons are associated with changes in Mn levels in the soil, the important variable was the season (72, 73). Soil Mn content was directly correlated with iron-magnesium minerals, and, except for very humid soils, most of the Mn in the native rocks is retained by the soil, and the Mn variations depending on soil depth were affected by soil pH and iron (74). The values of Mn metal in this study were in the range of 0.20–8.80 ppm compared to the global average, shale, and earth crust (850, 850, and 950 ppm, respectively), which is reported to be much lower (75). Given that most Iranian soils are alkaline and calcareous, the absorption efficiency of Mn elements in Iranian soils was very low. Many divalent metal cations (Mn, Fe, Co, Ni, Cu, and Zn) were structurally similar and replaced each other, causing soil disturbance (76, 77).

The Se content of the soil was higher at the third station than at the first and second stations. In winter, soil Se content was higher in the stations without vegetation cover than in the stations with vegetation cover, but in summer it was lower in the stations without vegetation cover compared to the stations with vegetation cover. Se is one of the most distributed elements throughout the Earth at 0.1–2 ppm depending on the geographical area (78). Accordingly, the amount of Se in the coastal soils of Shadegan wetland was higher than the normal range. The average Se content (existence or lack) in 135 soil samples of (79), depends on the content of soil parent materials, soil leaching, and secondary soil formation processes from plants (79). The amounts of Se in the soil in this study ranged from 1 to 4.25 ppm. In most soils of this province, the amount of Se was reported to be 3.7, which was higher than the natural values found in soil (0.1–2 ppm) (79), which confirms the results of this study. However, the amount of this element in the soils of China is found to be 0.14–0.37 ppm, which is inconsistent with the results of the present work (80). In some countries, such as New Zealand, Finland, and particular areas of China, the available Se in the soils is naturally low (81). The amount of soil Se depends on the composition of the bedrock from which the soil originates (82). The interaction between Se and other nutrients, such as phosphate, reduces the availability of Se in the soil, thereby reducing the uptake and accumulation of Se in the plant (83). Se content in soils also depends on the geological conditions, which can be made available to plants. Sandy soils have less Se content compared to organic and calcareous soils (84, 85). It is very important to have elements like Se in the soil, since plants take elements from the soil solution. Se is important for the growth and development of humans and animals, and also for plants in the soil, as they would absorb Se and utilize it for growth (86, 87). However, it should be noted that Se in high concentrations is toxic to living organisms, and the toxicity of this metal has been reported at a limited range of levels (88).

The Mo levels in both the winter and summer seasons at all the studied stations without vegetation cover were observed to be higher than the stations with vegetation cover. Mo is one of the metals that show the effects of ecological toxicity reactions (89). The amount of Mo in this study was between 0.01 and 0.96 ppm compared to the global average, shale, and earth crust (2, 3, and 1.5 ppm), which were lower (85).

The contamination factor for Zn, Cu, Co, Mn, Se, and Mo was <1, indicating the low contamination of these metals in the soil. The CF for Se was in the range of 1–3, indicating moderate contamination for this metal. The CF for Se at the third station was 3.666, which exhibited the high contamination of this element. The contamination factor for heavy metals implies that the concentration of these elements is higher than that of the earth's crust and shale. As Se levels show moderate to high levels of contamination, this element appears to have come from human activities in the coastal soils of Shadegan wetland. Se is one of the most abundant elements in the environment and also one of the most important constituents of soil (90).

The values of Cdeg for heavy metals in the coastal soils of Shadegan wetland were in the range of 1.296–1.845, and according to the classification of this index, values <7 indicate low contamination. In this study, the Cdeg for heavy metals was <7. Herein, the level of mCdeg was <1, which indicates very low contamination according to the classification of this index (values <1.5). Moreover, as the pollution load index values for heavy metals in the coastal soil of Shadegan wetland were <1, low contamination of these elements was observed. The values of the PLI vary from zero (non-contaminated) to 10 (highly contaminated). Typically, values <1 indicate non-contamination, and values >1 indicate contamination with heavy metals (50).

According to the results, the EF revealed low levels of pollution for zinc, copper, cobalt, selenium, nickel, manganese, and molybdenum, which seems to be due to the accumulation of geological and natural activities in the region. Cadmium had a significant EF in the soil, which showed that the amount of this metal in the coastal soils of Shadegan wetland was anthropogenic.

In a study on heavy metals in the Huixian wetlands in southern China, Huang et al. reported that there are moderate to high ecological hazards due to heavy metals in the coastal soils of this wetland (91). The concentrations of all the elements present in the Huixian wetland were observed to be greater than the background values. According to another study, China's Yellow River wetlands were contaminated with arsenic and Cd metals, and no contamination was observed due to chromium, Cu, Ni, lead, and Zn. However, the values of the pollution index demonstrated a low level of pollution for all the samples (12). Since the ecological risk of all the studied metals, including Zn, Cu, Co, Mn, Se, and Mo, was lower than 40 for all the studied stations in winter and summer, Zn, Cu, Co, Mn, Se, and Mo were in the low-risk classification. Furthermore, the risk index (RI) potential of metals was found to be in the range of 1.296–3.845, which is much lower than 150, and therefore the ecological risk potential of the studied metals was observed to be in the low-risk classification.

## Conclusion

The purpose of this study was to investigate the ecological risk assessment and pollution load index of toxic elements (zinc, copper, cobalt, molybdenum, manganese, and selenium) caused by the activity of agricultural fertilizers, vehicles, and industries in the coastal soils of southwest Iran.

According to our findings, the EF illustrated low levels of pollution for Zn, Cu, Co, Se, Mn, and Mo, which seems to be in accordance with the accumulation of geological and natural activities in the region. In other words, PLI, Cdeg, and mCdeg levels of pollution justify the low levels of metal contamination in the soil.

It seems that Zn, Cu, Co, Se, and Mo could be the result of human activities, and industrial and agricultural activities have played an effective role in the accumulation of these metals in the soil, but the main source of Mn metal is believed to be natural and geological. In both winter and summer, the ER values for Zn, Cu, Co, Mn, Se, and Mo were obtained from fewer than 40 stations; thus, Zn, Cu, Co, Mn, Se, and Mo levels were low. Heavy metal health risk assessment (RI) in the studied coastal soils showed that the metals like cadmium, molybdenum, copper, selenium, zinc, manganese, and cobalt were found to be at low risk and do not pose a particular problem for human health.

## Data availability statement

The original contributions presented in the study are included in the article/Supplementary material, further inquiries can be directed to the corresponding author.

## Ethics statement

Getting informed consent is not required for this study because the data were collected through observation, and there were no human participants. The Ethics Committee of the Islamic Azad University of Ahvaz Branch confirmed that the study was moral and ethical.

## Author contributions

EH, KH-P, MT-K, and NS were principal investigators of the study, drafted the manuscript, advisors on the study, and performed the statistical analysis. All authors contributed to the design, data analysis, assisted in the preparation of the final version of the manuscript, read, and approved the final version of the manuscript.

## Funding

The authors are grateful to the Islamic Azad University of Ahvaz Branch for providing the necessary facilities to perform this research.

## Conflict of interest

The authors declare that the research was conducted in the absence of any commercial or financial relationships that could be construed as a potential conflict of interest.

## Publisher's note

All claims expressed in this article are solely those of the authors and do not necessarily represent those of their affiliated organizations, or those of the publisher, the editors and the reviewers. Any product that may be evaluated in this article, or claim that may be made by its manufacturer, is not guaranteed or endorsed by the publisher.
