# Supplementary material for: Potential ecological risk assessment of heavy metals (trace elements) in coastal soils of southwest Iran
Source: Front Public Health. 2022 Sep 7;10:889130. doi: 10.3389/fpubh.2022.889130 (PMC9491490; doi:10.3389/fpubh.2022.889130)
Supplement: Supplementary file 1 [file Data_Sheet_1.docx]

**Potential ecological risk assessment of heavy metals (trace elements) in coastal soils of southwest Iran**

**Ebtessam Hamid^1^, Khoshnaz Payandeh^1*^, Mohammad Tahsin Karimi Nezhad^2^, Naghmeh Saadati^1^**

1. Department of Soil Science, Ahvaz Branch, Islamic Azad University, Ahvaz, Iran

2. Department of Agriculture and Plant Breeding, Sanandaj Branch, Islamic Azad University, Sanandaj, Iran

**Table S1:** Classification of soil pollution by contamination factor, contamination degree

| Classification | Contamination factor | Contamination degree |
| --- | --- | --- |
| Low pollution | CF < 1 | Cdeg< 7 |
| Medium pollution | CF < 3 ≥ 1 | Cdeg< 14 ≥ 7 |
| High pollution | CF < 6 ≥ 3 | Cdeg< 28 ≥ 14 |
| Extremely polluted | CF ≥ 6 | Cdeg ≥ 28 |

**Table S2:** Describing environmental pollution based on modified contamination degree

| Quality of the studied environment | Modified contamination degree values |
| --- | --- |
| Very low levels of contamination | Cdeg<1.5 |
| Low levels of contamination | 1.5 <Cdeg<2 |
| Average levels of contamination | 2 <Cdeg<4 |
| High levels of contamination | 4 <Cdeg<8 |
| Very high levels of contamination | 8 <Cdeg<16 |
| Severe levels of contamination | 16 <Cdeg<32 |
| Infinitely high levels of contamination | Cdeg>32 |

**Table S3:** Classes of biological hazard and ecological risk of heavy metals

| RI value | Amount of ecological risk | Er value | Classes of biological hazard |
| --- | --- | --- | --- |
| RI <150 | Low | < 40 Er | Low |
| RI < 300 ≥150 | Medium | < 80 Er≥40 | Medium |
| RI < 600 ≥300 | Extreme | < 160Er≥80 | High |
| RI ≥ 600 | Very extreme | < 320Er≥160 | Extreme |
| - | - | ≥ 320 Er | Very extreme |

**Table S4:** Enrichment factor in samples

| **Enrichment intensity** | **Enrichment factor** |
| --- | --- |
| No enrichment | EF≤ 1 |
| Low enrichment | 1< EF< 3 |
| Moderate enrichment | 3< EF< 5 |
| Relatively rich enrichment | 5< EF< 10 |
| Severe enrichment | 10< EF< 25 |
| Very severe enrichment | 25< EF< 50 |
| Extremely rich enrichment | EF> 50 |

**Table S5.** Matrix of Spearman's correlation coefficient for winter-collected coastal soils of Shadegan wetland, upper triangle

| **Variables** | **Cu** | **Co** | **Mn** | **Se** | **Silt** | **Clay** | **Sand** | **pH** | **EC** | **SOC** |
| --- | --- | --- | --- | --- | --- | --- | --- | --- | --- | --- |
| Zn | -0.164 | -0.266 | 0.551* | -0.441 | 0.190 | -0.048 | -0.156 | -0.507* | 0.502* | 0.201 |
| Cu |  | 0.609** | 0.002 | 0.213 | -0.373 | 0.354 | 0.251 | -0.008 | -0.067 | -0.381 |
| Co |  |  | 0.111 | 0.229 | -0.117 | -0.047 | 0.184 | -0.048 | -0.252 | -0.246 |
| Mn |  |  |  | -0.067 | 0.093 | -0.072 | -0.011 | -0.676** | 0.470 | 0.335 |
| Se |  |  |  |  | 0.137 | 0.089 | -0.322 | -0.142 | 0.177 | -0.479* |
| Silt |  |  |  |  |  | -0.718*** | -0.792*** | -0.062 | 0.377 | -0.080 |
| Clay |  |  |  |  |  |  | 0.215 | -0.191 | 0.027 | -0.005 |
| Sand |  |  |  |  |  |  |  | 0.210 | -0.502* | 0.150 |
| pH |  |  |  |  |  |  |  |  | -0.495* | -0.159 |
| EC |  |  |  |  |  |  |  |  |  | -0.230 |

*: P<0.05; **:P<0.01: ***:P<0.001

**Table S6.** Matrix of Spearman's correlation coefficient for summer-collected coastal soils of Shadegan wetland, upper triangle

| **Variables** | **Cu** | **Co** | **Mn** | **Se** | **Silt** | **Clay** | **Sand** | **pH** | **EC** | **SOC** |
| --- | --- | --- | --- | --- | --- | --- | --- | --- | --- | --- |
| Zn | 0.014 | 0.022 | 0.179 | -0.697** | -0.394 | -0.692** | 0.705*** | 0.157 | 0.179 | -0.345 |
| Cu |  | 0.291 | 0.260 | 0.158 | 0.212 | 0.025 | -0.097 | -0.009 | 0.316 | 0.280 |
| Co |  |  | 0.409 | -0.161 | -0.065 | -0.065 | 0.276 | 0.492* | -0.330 | -0.282 |
| Mn |  |  |  | -0.086 | -0.027 | -0.414 | 0.397 | -0.296 | 0.064 | 0.368 |
| Se |  |  |  |  | 0.430 | 0.454 | -0.466 | -0.196 | -0.182 | 0.453 |
| Silt |  |  |  |  |  | 0.237 | -0.475* | -0.205 | 0.090 | 0.400 |
| Clay |  |  |  |  |  |  | -0.792*** | -0.108 | 0.042 | 0.190 |
| Sand |  |  |  |  |  |  |  | 0.379 | -0.121 | -0.363 |
| pH |  |  |  |  |  |  |  |  | -0.505* | -0.743*** |
| Variables | Cu | Co | Mn | Se | Silt | Clay | Sand | pH | EC | SOC |

*: P<0.05; **:P<0.01: ***:P<0.001
